# Cerebral Venous Thrombosis Following Periocular Filler Injection—A Case Report and Review of Literature

**DOI:** 10.1155/crnm/9363655

**Published:** 2025-09-03

**Authors:** Reza Asgari, Mohammadamin Bazzazan, Fateme Jafari, Hossein Mozhdehipanah

**Affiliations:** ^1^Student Research Committee, Qazvin University of Medical Sciences, Qazvin, Iran; ^2^Department of Neurology, Bou Ali Sina Hospital, Qazvin University of Medical Sciences, Qazvin, Iran

**Keywords:** CVT, filler complication, hyaluronic acid, periocular injection, seizure

## Abstract

**Purpose:** To evaluate the effect of periocular filler injection on the incidence of cerebral venous thrombosis (CVT).

**Case Report:** A 41-year-old woman without a prior medical history experienced a severe headache and subsequent seizures following an eye filler injection. Diagnosis of CVT was confirmed through brain magnetic resonance venography (MRV), revealing thrombosis in the left transverse and upper sagittal sinuses. The patient was treated with intravenous heparin and oral warfarin, leading to improvement and discharge in good condition after 10 days of hospitalization.

**Conclusion:** This case underscores the potential risk of CVT following periocular filler injections, emphasizing the need for awareness and preventive measures among medical professionals.

## 1. Introduction

Cerebral venous sinus thrombosis (CVST) is a rare disorder with an estimated annual incidence of 3 to 4 cases per million overall and an annual incidence of 12.3 per million in Iran, which occurs when a blood clot forms in the brain's venous sinuses [1]. The clot keeps blood from draining out of the brain. As a result, pressure builds up in the blood vessels. This can lead to swelling and bleeding (hemorrhage) in the brain. Despite its rarity, timely diagnosis is crucial, as it can lead to severe neurologic complications including seizures, cerebral edema, and venous infarction [2].

This chain of events is part of a stroke that can occur in adults and children. It can occur even in newborns and babies in the womb. A stroke can damage the brain and central nervous system. A stroke is serious and needs medical attention right away [2].

Many risk factors contribute to the development of cerebral venous thrombosis (CVT). At least one risk factor was identified in more than 85% of patients with CVT, and multiple risk factors were found in more than 50% of patients with CVT. In general, CVT is common in any condition that leads to a prothrombotic state, including pregnancy, the postpartum state, or those on oral contraceptives. In the International Study on Cerebral Vein and Dural Sinus Thrombosis (ICSVT), genetic and acquired thrombophilia were present in 34% of patients with CVT. Inherited thrombophilia includes protein C and protein S deficiencies, antithrombin deficiency, factor V Leiden mutation, prothrombin gene mutation 20,210, and hyperhomocysteinemia [3].

Patients with hereditary thrombophilia have an increased predisposition for developing any form of thrombosis, including CSVT. G20210A prothrombin polymorphism, factor V Leiden, and antiphospholipid syndrome are the most frequent causes. Protein C, S deficiency, and antithrombin III are less commonly encountered risk factors [4].

The clinical course of CVST varies widely—some patients have mild symptoms and recover fully, while others may rapidly deteriorate and suffer severe disability or even death. Although around 90% of patients regain functional independence, nearly 40% still report ongoing symptoms such as headache, fatigue, or cognitive and emotional disturbances 6 months after the event, which can limit their ability to resume normal activities. The overall mortality rate is about 3%, with nearly half of the deaths associated with hemorrhagic complications [5].

**Figure 1:**
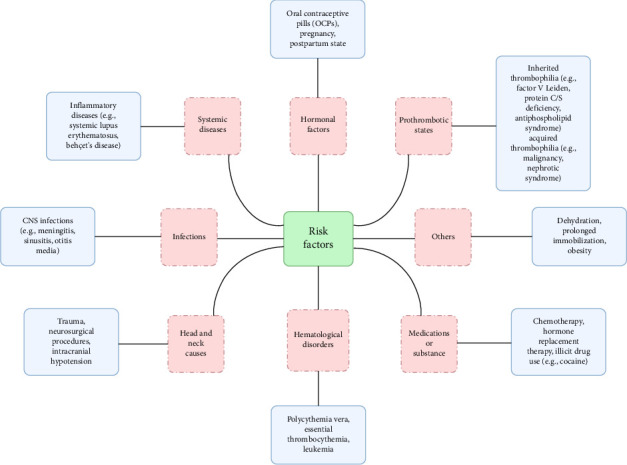
A comprehensive diagram eliciting risk factors associated with CVT.

A wide variety of precipitating factors have been described, but cosmetic procedures are not commonly listed among them. The increasing popularity of facial filler injections, particularly hyaluronic acid (HA)-based products, has led to growing concern about their potential vascular complications. While most reports focus on arterial occlusion and immediate visual or neurologic symptoms, CVT as a delayed complication of filler injection is exceedingly rare. All risk factors associated with CVT are listed in Table 1 and Figure 1 [6].

Here, we present the case of a previously healthy 41-year-old woman who developed CVT 10 days after a periocular filler injection. This case highlights an unusual and delayed presentation of a potentially life-threatening vascular complication and underscores the need for heightened awareness among clinicians and aesthetic practitioners.

## 2. Case Presentation

The patient is a 41-year-old woman with no past medical history and no history of smoking, alcohol consumption, or recreational drug use or other risk factors for CVT, such as OCP consumption, who presented with a complaint of convulsions. She states that about 10 days ago, following the injection of eye filler in the periorbital area, she had a very severe headache, which she had never experienced before. She was unaware of the exact aesthetic procedure done for her, unfortunately. The headache in the frontal area was bilateral with a pressing nature and did not respond to painkillers. She did not have blurred vision, double vision, or dizziness. At noon on the day of the visit, they first had a GTC-type seizure that lasted for 2-3 min and was repeated 6 h later in the emergency room. The second seizure was quickly controlled with diazepam.

The patient appeared lethargic but arousable, consistent with a postictal state. The patient was oriented to place, time, and person. Speech was fluent when alert, without dysarthria or aphasia. No signs of meningeal irritation (no neck stiffness or photophobia) were observed. The patient had no fever. Pupils were equal, round, and reactive to light bilaterally. Extraocular movements were intact, with no nystagmus or diplopia. Visual fields were grossly intact to confrontation. Fundoscopic examination of the right eye revealed blurring of the nasal, superior, and inferior margins of the optic disc, suggestive of papilledema. No facial asymmetry was noted. Hearing was intact bilaterally. Palate elevation and tongue movements were midline. The strength of the right limbs was +4.5 during the examination. Plantar reflexes were flexor bilaterally. Gait was initially assessable and normal.

Light touch, pinprick, and proprioception were preserved in all extremities. Finger-to-nose and heel-to-shin testing were intact on the left and mildly slowed on the right. No dysmetria was observed.

### 2.1. Lab Test

During hospitalization, the patient's headache gradually improved. Also, the patient's seizure was controlled. At first, intravenous heparin was started for her, and oral warfarin was added following PTT disturbance. It was the protocol of the institution to start unfractionated heparin for these patients and it was also preferred due to its short half-life, rapid reversibility, and the ability to closely monitor and adjust dosing via aPTT. This was important given the patient's neurologic instability and the risk of hemorrhagic complications associated with increased intracranial pressure. Although there is a subtle drop in PTT, it returned to the expected level after the first day of hospitalization. The PTT levels in the following table were for the patient's first day of hospitalization. The patient experienced a mild and transient thrombocytopenia, which subsequently returned to normal levels and was not clinically significant. The patient's important lab results are included in Table 2.

After 10 days of hospitalization in this center, the patient was discharged with good general condition, oral warfarin, and a recommendation to perform coagulation tests on an outpatient basis.

### 2.2. Imaging Findings

#### 2.2.1. Brain CT

**Figure 2:**
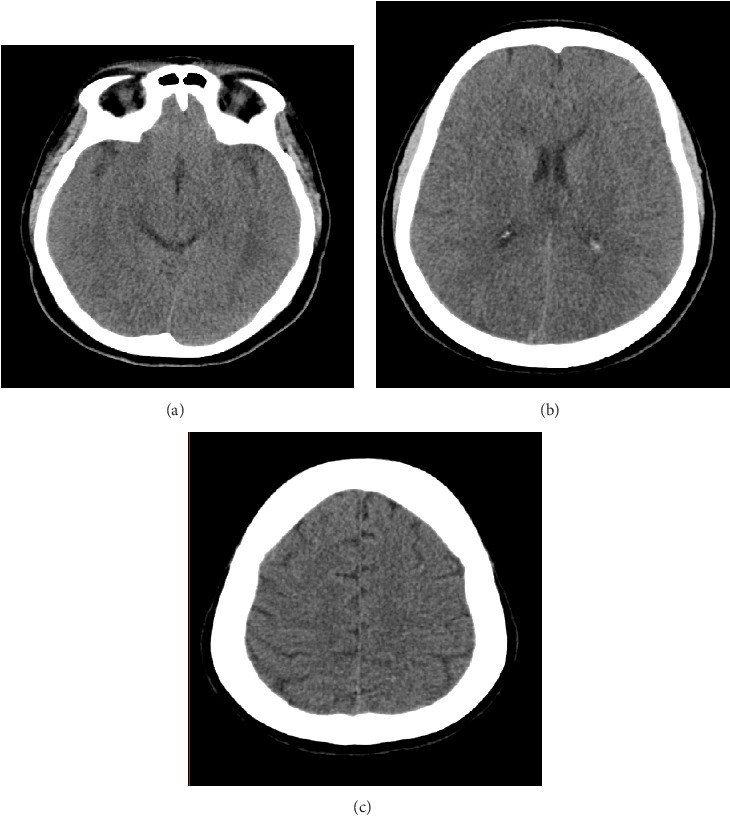
Sections of the patient's head CT scan without any significant findings.

Noncontrast brain CT images, included in Figures 2(a), 2(b), and 2(c), showed no evidence of acute lesions such as infarction, hemorrhage, or mass effect. Subtle findings suggestive of mild cerebral swelling, such as partial effacement of the cortical sulci, were noted in certain cuts; however, these changes were not uniform. In other sections of the scan, the sulci remained visible, and there was no significant midline shift or ventricular compression. Overall, the imaging did not demonstrate any definitive signs of acute pathology.

#### 2.2.2. Brain MRI

**Figure 3:**
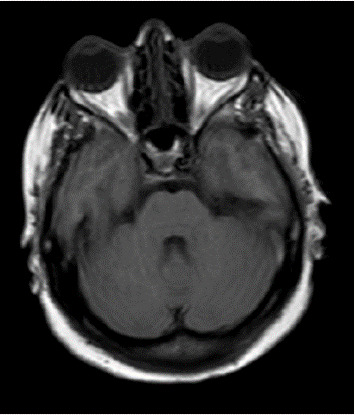
A section of the patient's T1-weighted MRI scan.

**Figure 4:**
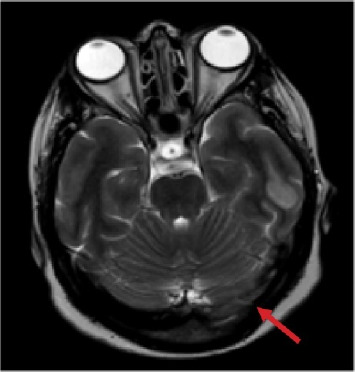
T2-weighted MRI scan demonstrating a signal abnormality in the left transverse (lateral) sinus, consistent with sinus thrombosis, which is marked by an arrow.

**Figure 5:**
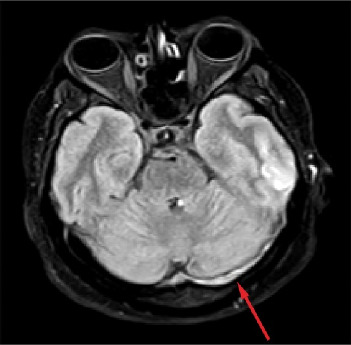
FLAIR MRI scan showing evidence of a cortical infarction in the left temporal lobe, visible as hyperintensity in the affected region and signal abnormality in the left transverse (lateral) sinus, consistent with sinus thrombosis, which is marked by an arrow.

Figure 3 (T1-weighted) appears unremarkable. In Figure 4 (T2-weighted) and Figure 5 (flair), there is evidence of a cortical infarction in the left temporal lobe, seen as hyperintensity in the affected region. Additionally, both figures demonstrate a signal abnormality in the left transverse (lateral) sinus, consistent with sinus thrombosis, which is marked by an arrow. These findings support the diagnosis of CVT with associated venous infarction.

#### 2.2.3. Brain Magnetic Resonance Venography (MRV)

**Figure 6:**
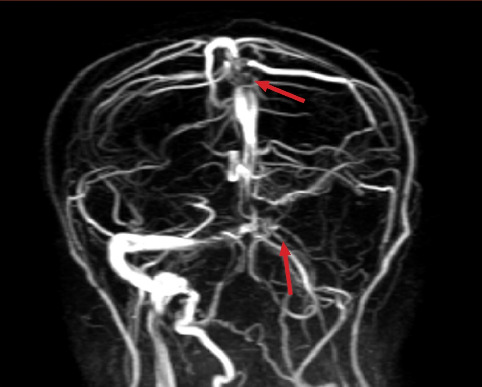
MRV image indicating evidence of thrombosis in the left transverse and upper sagittal sinus (pointed at with red arrows).

Evidence of thrombosis was seen in the left transverse and upper sagittal sinus in Figure 6 (pointed at with red arrows).

## 3. Discussion and Review of Literature

As mentioned, CVT is one of the incidents that can occur under various circumstances. Genetic factors such as hereditary coagulopathy or environmental factors such as using OCP may serve as its etiology. One of the common procedures, especially among women, is the use of fillers for facial contouring. The term “filler” refers to the use of any volumizing substance through injection. Typically, fillers come in various types based on the substance used, including diverse materials such as HA products, collagen, paraffin, and liquid silicon [2]. Using a filler can have various spectrums of complications, but the important ones are vascular ischemia and tissue necrosis [3]. Based on the proposed mechanisms for the problems caused by filler injection and its significant association with CVT, it can be considered one of the etiologies of thrombosis in brain vessels and important facial vessels [7]. In the following, we reviewed the reported cases like the one under consideration in this article.

In 2013, He et al. reported a 52-year-old female patient with the injection of HA in glabella [8]. It was stated in the report that the patient experienced sudden eye pain, headache, and vision loss shortly after receiving a HA injection in the glabellar area. She was diagnosed with central retinal artery occlusion and brain infarction, leading to blindness in her right eye and left hemianopia. Despite treatment, her vision did not recover, and follow-up revealed continued retinal ischemia and emboli in the right eye. In 2014, Hong et al. reported a 50-year-old female patient with cosmetic facial injections that contained HA in the site of glabella and cheeks [9]. Occlusion in the left anterior and middle cerebral arteries, and the left central retinal artery was reported shortly after the injection. Right hemiparesis and facial palsy left eye ptosis and blindness were the follow-up results. In 2014, Kim et al. presented a 23-year-old male patient with an injection of HA into the nose [10]. Infarction in the right frontotemporoparietal lobes and occlusion in the right ophthalmic artery were reported shortly after the injection. Right eye blindness, right facial palsy, and left limb paralysis were the result of follow-up. In 2015, Lin et al. reported a 25-year-old female patient with a nasal filler injection [11]. The patient experienced severe eye pain, nausea, and dizziness, followed by loss of vision in her right eye 3 hours after receiving a nasal HA filler injection. Examination revealed right eye ptosis, no light reflex, and signs of central retinal artery occlusion. Later, she developed decreased muscle power in her left arm. Brain MRI showed multiple small acute infarcts, primarily in the middle cerebral artery territory, and reduced flow in the right ophthalmic artery. In 2015, Li et al. reported a delayed-onset cerebral infarction in a 25-year-old woman with the nasal injection of HA [11]. A patient with no known systemic diseases, experienced sudden right eye blindness after a nasal HA injection, followed by left upper limb weakness 9 hours later. Initially diagnosed with right central retinal artery occlusion, her condition worsened, leading to left upper limb weakness with muscle power graded 4. Brain MRI revealed multiple small acute infarcts, primarily in the right middle cerebral artery territory. A transesophageal echocardiogram identified a 2.7 cm atrial septal defect. Despite treatment, her vision loss and limb weakness persisted after discharge. In 2018, Ansari et al. reported ocular and cerebral infarction in a 20-year-old female patient with a periocular filler injection [12]. The patient experienced vision loss in her right eye and skin necrosis on her nose after receiving an HA filler injection in the glabella 1 month earlier. She had no light perception in her right eye, and an examination revealed a fibrotic membrane from a pale optic nerve and an atrophic retina. MRI showed scattered infarcts in the right parietal lobe. These findings indicated that the filler was inadvertently injected into the supraorbital artery, leading to occlusion of the right ophthalmic artery and other arteries, demonstrating the risks of vascular complications in cosmetic procedures. In 2019, Yang et al. reported a 36-year-old female patient with a 2 mL nasal HA injection [13]. The injection caused infarction in both the right and the left frontal and temporal lobes and left parietal and occipital lobes. Also, occlusion was reported in the left ophthalmic artery. Because of the complicated situation, the patient, in the follow-up, died. In 2020, Tao et al. presented a 22-year-old female patient with an injection of HA in her left temporal and forehead, and the chief complaint was the development of headache, vertigo, nausea, and blindness of her left eye after injection [14]. The patient experienced left eye blindness, vertigo, and nausea 10 min after a HA injection. By the next day, she developed hemiplegia and was found to have intracranial hemorrhage and thrombosis in the left sigmoid venous sinus. Despite treatment with anticoagulants and other medications, her left eye vision remained impaired at discharge on April 11, though her other symptoms improved. This case highlights the severe risks and timeline of complications following HA injections. In 2021, Eldweik presented a 32-year-old female patient with orbital infarction after injection of HA over the nasal bridge [15]. The patient lost vision in her left eye seconds after receiving a 0.5 mL HA filler injection during a rhinoplasty. Despite immediate hyaluronidase treatment, she developed swelling and bluish discoloration. Within an hour, her vision was evaluated as no light perception, and she was diagnosed with acute left orbital infarction. Further treatment, including peribulbar hyaluronidase injections and steroids, showed no improvement in vision. After 8 weeks, her vision remained lost, though eye movement and ptosis improved, with residual scarring and severe ischemia of the optic nerve and retina.

Additionally, there was a case involving a 46-year-old female who underwent an HA injection into the superior palpebral region under local anesthesia. She presented symptoms of an emotional disorder characterized by hyperactivity, along with experiencing vision loss in her right eye 3 hours after the injection. Neurological imaging revealed the presence of a lacunar cerebral infarction. Treatment included the administration of glucocorticoids, neurotrophic drugs, and hyperbaric oxygen, yet there was no notable improvement observed in her condition [16].

In previous instances of related cases, HA was administered via injection to periorbital regions, such as the nose, for noninvasive rhinoplasty [13, 16], the glabella [12, 17], and the forehead [17]. Most of these cases had no prior health issues. Patients experienced various complications, including but not limited to vision impairment, headache, nausea, weakness, dizziness, dysarthria, hemiplegia, facial paralysis, and emotional or consciousness disorders. The onset of these complications may vary, occurring either during or several hours after the operation. In most cases, emboli tend to occur in locations such as the middle cerebral artery and anterior cerebral artery, resulting in infarction. Patients underwent interventions for vascular blockages; some of them received thrombolytic therapy for clot dissolution. Some of them underwent decompressive craniectomy procedures. Furthermore, antiplatelet/anticoagulant therapy was administered to a number of patients. The remaining individuals were managed with symptomatic and nutritional modalities, encompassing the use of steroids, nutritional neuropharmaceuticals, mannitol, and hyperbaric oxygen. Predominantly, the surgical intervention for vascular blockages emerged as the most frequently employed treatment modality.

Compared to previously reported cases, our patient's presentation is notably atypical in several ways. Most documented complications following HA filler injection occur within minutes to a few hours and involve arterial occlusion, primarily affecting the ophthalmic artery or branches of the anterior and middle cerebral arteries. These cases typically present with sudden visual loss, hemiparesis, or altered consciousness shortly after injection, as seen in reports by He et al., Hong et al., and Ansari et al. [8, 9, 12]. In contrast, our patient presented 10 days after a periocular injection, with a progressive headache followed by seizures and no visual symptoms, suggesting a different pathophysiological mechanism. Imaging revealed a cortical infarct in the left temporal lobe and left lateral sinus thrombosis, findings not characteristic of arterial embolism but rather of CVT. Among the reviewed literature, only the case by Tao et al. involved venous sinus thrombosis after filler injection, though that patient experienced hemorrhagic transformation, visual loss, and symptoms within 24 h [14]. Our case, while similar in mechanism, is unique in its delayed onset, absence of ocular involvement, and isolated venous infarction without hemorrhage. These distinctions support the hypothesis that in rare instances, filler material may enter superficial facial veins, eventually propagating into cerebral venous structures and causing delayed thrombosis, even in patients without underlying prothrombotic risk factors. This case underscores the importance of considering CVT in patients with delayed neurologic symptoms following facial filler procedures, especially when injections are administered in high-risk anatomical regions such as the periorbital and temporal areas.

Several clinical and contextual features in this case support the hypothesis that the CVT was induced by the periocular filler injection. First, the patient was previously healthy and lacked traditional prothrombotic risk factors such as thrombophilia, malignancy, or hormonal therapy. Second, there was a clear temporal relationship between the injection and the onset of symptoms, with a new, severe headache beginning shortly after the procedure and progressing to seizures 10 days later. Third, imaging demonstrated thrombosis in the left transverse sinus—a venous territory potentially affected by retrograde embolization from superficial facial veins, particularly given the anatomical proximity to the periorbital region. Lastly, similar filler-associated venous thrombosis cases have been documented in the literature, reinforcing the biological plausibility of this mechanism.

On the other hand, the delayed onset of symptoms (10 days postinjection) raises some uncertainty regarding a direct causal link, as most vascular filler complications tend to manifest acutely, often within hours. Additionally, no direct evidence of intravascular filler material was obtained through imaging or biopsy. Despite these limitations, in the absence of alternative explanations and in light of the anatomical and temporal context, a filler-induced CVT remains the most plausible diagnosis.

There are 3 main mechanisms proposed for explaining cerebral and ocular embolization after HA injection. The first mechanism is that the filler material enters extracranial vessels of the ophthalmic artery due to injured vessels as a result of high pressure injection which then can occlude both the middle cerebral artery and the ophthalmic artery and immediately cause symptoms. The second scenario is when the pressure of injection causes injury to superficial temporal artery and pushes filler material all the way back to the common carotid, from which it can enter the internal carotid artery and occlude the middle cerebral artery and the ophthalmic artery and immediately cause problems. The third proposed route is when the filler material enters superficial facial veins and is pushed anterogradely to the heart, and from there it can cause brain, lung, and eye emboli in a delayed manner. In Table 3, there is a brief explanation of each similar case included in the discussion and their proposed mechanism for thrombosis formation.

Complications from facial filler injections are rare but potentially serious, including vascular thrombosis and cerebral embolism. Due to limited treatment options, prevention is essential. Recommended safety measures include slow injection of small volumes, use of blunt cannulas, aspiration before injection, and ensuring the procedure is performed by skilled professionals. In case of vascular events, immediate management with hyaluronidase, aspirin, corticosteroids, vasodilators, and anticoagulants is advised, along with prompt referral if visual or neurologic symptoms occur [19, 20].

## 4. Conclusion

This case highlights a rare but serious delayed complication of HA filler injection—CVT. Unlike most previously reported vascular filler complications, which involve acute arterial occlusion, our patient presented 10 days after periocular injection with venous sinus thrombosis and no other identifiable prothrombotic risk factors. The anatomical proximity of the injection site to valveless facial veins that communicate with intracranial venous sinuses provides a plausible route for embolic or inflammatory propagation. Furthermore, the clear temporal association, the absence of alternative causes, and known biological mechanisms support a likely connection between the procedure and the thrombotic event [4].

While the rarity of CVT following filler injection limits definitive conclusions, and only a single similar case has been reported, the overall clinical context makes a causal relationship highly plausible. We estimate the probability of the filler injection being the precipitating factor in this case to be high [13].

This case underscores the need for increased awareness among aesthetic and medical professionals regarding the potential for delayed cerebral venous complications following facial filler use, particularly in high-risk anatomical regions. Preventive technique, thorough patient evaluation, and early recognition of neurologic symptoms remain key to minimizing risk.

## Figures and Tables

**Table 1:** Risk factors associated with CVT.

Category	Risk factors
Prothrombotic states	Inherited thrombophilia (e.g., factor V Leiden, protein C/S deficiency, and antiphospholipid syndrome)
	Acquired thrombophilia (e.g., malignancy and nephrotic syndrome)
Hormonal factors	Oral contraceptive pills (OCPs), pregnancy, postpartum state
Systemic diseases	Inflammatory diseases (e.g., systemic lupus erythematosus, and Behçet's disease)
Infections	CNS infections (e.g., meningitis, sinusitis, and otitis media)
Head and neck causes	Trauma, neurosurgical procedures, intracranial hypotension
Hematological disorders	Polycythemia vera, essential thrombocythemia, and leukemia
Medications/Substances	Chemotherapy, hormone replacement therapy, and illicit drug use (e.g., cocaine)
Others	Dehydration, prolonged immobilization, and obesity

**Table 2:** Important lab results of the patient.

WBC	7.6 × 10^3^/μL				
Hb	12.3 g/dL				
Plt	140 × 10^3^/μL				
Bs	118 mg/dL				
BUN	16 mg/dL				
Cr	0.9 mg/dL				
ESR	14 mm/hr				
INR	1	1.85			
PTT (s)	26	26.69	39.9	43	32.1
CPK	60 mg/dL				
Na	137 mEq/L				
K	4 mEq/L				
Ca	8.6 mg/dL				
P	1.7 mg/dL				
AST	12 U/L				
ALT	18 U/L				
ALP	172 U/L				

**Table 3:** A brief summary of similar cases included in the article.

Author	Key clinical features	Proposed mechanism	Outcome
He et al. [8]	52F, HA injection in glabella ⟶ sudden eye pain, headache, vision loss ⟶ central retinal artery occlusion + brain infarct	Retrograde arterial embolism via ophthalmic artery	Right eye blindness, left hemianopia, persistent deficits
Hong et al. [9]	50F, HA injection in glabella/cheeks ⟶ occlusion in anterior cerebral artery/middle cerebral artery + central retinal artery ⟶ hemiparesis, facial palsy, blindness	Arterial embolization through ophthalmic artery	Left eye blindness, persistent right hemiparesis
Kim et al. [10]	23M, nasal HA injection ⟶ infarction in frontal/temporal lobes + ophthalmic artery occlusion	High-pressure injection causing arterial emboli	Right eye blindness, facial palsy, left limb paralysis
Lin et al. [11]	25F, nasal HA injection ⟶ central retinal artery + middle cerebral artery infarcts ⟶ ptosis, vision loss, left arm weakness	Retrograde arterial embolism via ophthalmic artery	Persistent right eye blindness, mild hemiparesis
Li et al. [18]	25F, nasal HA ⟶ right eye blindness, upper limb weakness 9 h later ⟶ middle cerebral artery infarcts + atrial septal defect found	Embolism via patent foramen ovale (paradoxical embolism)	Persistent vision loss + limb weakness
Ansari et al. [12]	20F, periocular HA ⟶ right eye blindness, nasal skin necrosis, cerebral infarcts	Intravascular injection into supraorbital artery ⟶ ophthalmic artery occlusion	Right eye blindness, nasal tissue necrosis
Yang et al. [13]	36F, 2 mL nasal HA ⟶ infarcts in frontal, temporal, parietal, occipital lobes + ophthalmic artery occlusion	Extensive arterial embolization via facial/ophthalmic circulation	Death
Tao et al. [14]	22F, HA in forehead/temple ⟶ blindness, vertigo, then hemiplegia + sigmoid sinus thrombosis	Venous embolism via superficial facial veins ⟶ delayed CVT	Vision loss persisted; other symptoms improved
Eldweik [15]	32F, nasal bridge HA ⟶ vision loss within seconds, orbital infarction	Immediate arterial embolization via ophthalmic artery	Complete vision loss, residual ischemic damage
Zhang et al. [16]	46F, superior palpebral HA ⟶ lacunar infarction, emotional disorder, vision loss	Not explicitly stated; likely arterial embolism	No significant improvement with treatment

## Data Availability

The data that support the findings of this study are available on request from the corresponding author. The data are not publicly available due to privacy or ethical restrictions.
